# Drug-Induced Undifferentiated Connective Tissue Disease in a Two-Year-Old Girl: A Manifestation of Factitious Disorder Imposed on Another (FDIA)

**DOI:** 10.7759/cureus.60607

**Published:** 2024-05-19

**Authors:** Kelvin E Oppan, Stephanie Otupri

**Affiliations:** 1 Pediatrics, Ternopil National Medical University, Ternopil, UKR

**Keywords:** emergency medicine, child abuse, auto-inflammatory disease, undifferentiated connective tissue disease, munchausen syndrome by proxy, factitious disorder imposed on another

## Abstract

Factitious disorder imposed on another (FDIA), formerly known as Munchausen syndrome by proxy (MSBP), constitutes a form of child abuse wherein a caregiver fabricates or induces illness in a person under their care or supervision. Here, we present a case of a two-year-old girl with signs and symptoms suggestive of undifferentiated connective tissue disease (UCTD) and probable autoinflammatory disease, which was a manifestation of FDIA. The patient manifested recurrent febrile episodes and presented with hepatosplenomegaly, elevated inflammatory markers, and mesangial proliferative glomerulonephritis. Regardless of extensive medical interventions, including corticosteroids and immunosuppressive therapy, the patient's condition failed to improve until the caregiver was isolated from the patient. Upon questioning, the caregiver admitted to having administered pyrogenal, an immunomodulator, to induce symptoms. This case highlights the challenges and difficulties of diagnosing and managing FDIA-associated illnesses, drawing attention to the importance of considering this diagnosis in cases of unexplained or recurrent fever in children.

## Introduction

Factitious disorder imposed on another (FDIA), previously known as Munchausen syndrome by proxy (MSBP), is a severe form of child abuse wherein a caregiver fabricates or induces illness in a dependent individual, resulting in unnecessary medical interventions and severe harm [1]. Despite a mortality rate between 6% and 10% among victims, FDIA remains underdiagnosed [2]. Usually, these caregivers intentionally induce sickness in the victims for psychological gratification [3-5]. While FDIA is uncommon, it can have significant physical and psychological consequences for the victim. In the context of palliative care, the diagnosis and management of FDIA-associated illnesses can be particularly challenging due to the complexity of individual patients' medical needs. We present the case of drug-induced undifferentiated connective tissue disease in a two-year-old girl, believed to be a demonstration of FDIA, to underscore the complexities and ethical considerations involved in such cases.

## Case presentation

**Figure 1:**
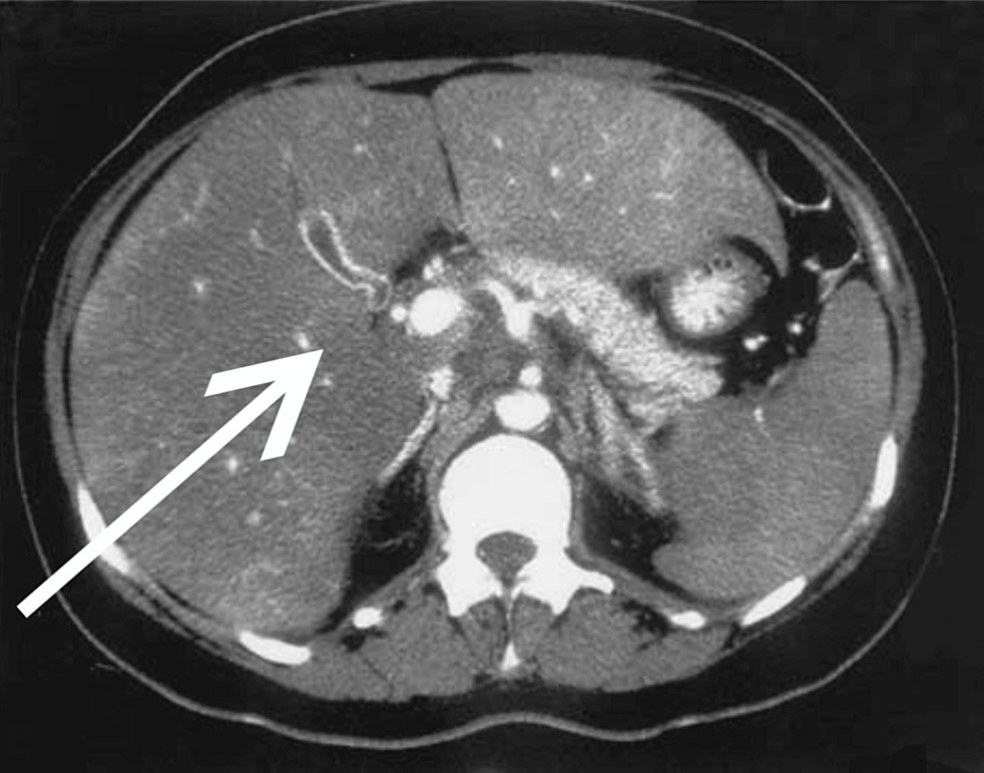
Abdominal CT showing diffuse hepatomegaly

**Figure 2:**
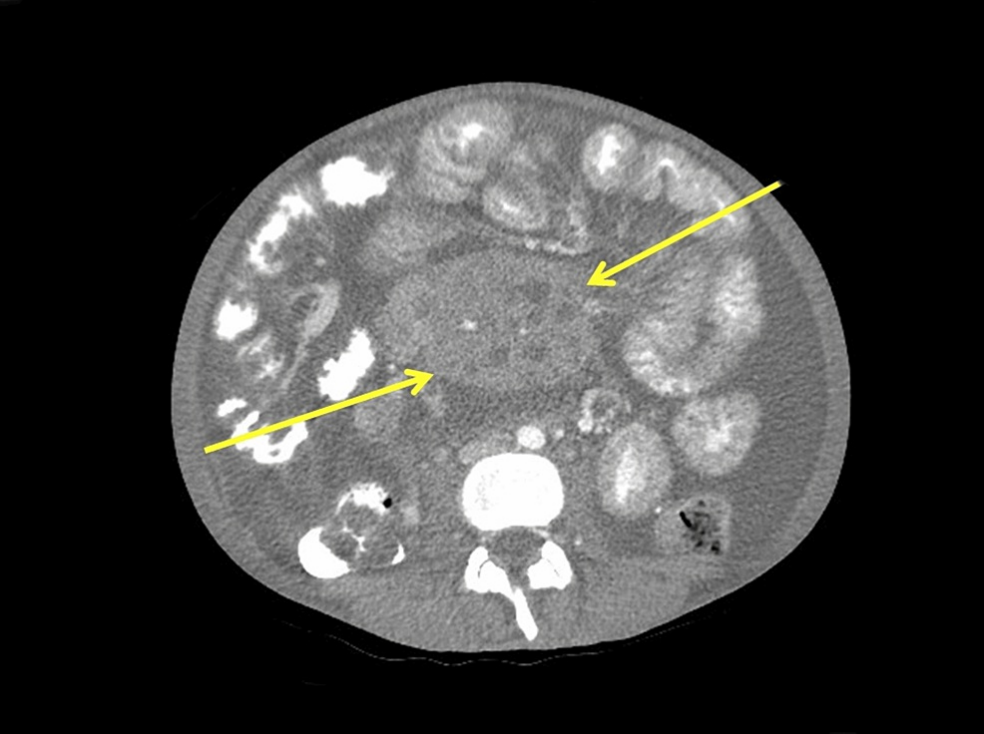
Abdominal CT showing mesenteric lymphadenopathy without splenomegaly

The patient is a two-year-old girl with symptoms of persistent febrile episodes for six months that were unresponsive to antipyretics, severe hepatosplenomegaly (Figure 1), mesenteric lymphadenopathy without splenomegaly (Figure 2), and lower back pain. Leukocytosis, anemia, high inflammatory markers (Table 1), and mesangial proliferative glomerulonephritis (Table 2) were all recorded. Despite interventions in treatment that included antibiotics, nonsteroidal anti-inflammatory drugs (NSAIDs), and detoxification therapy for an unknown intoxication syndrome, the patient's condition did not improve. Subsequently, the patient underwent appendectomy due to the manifestation of positive alarming symptoms like changes in bowel habits, nausea and vomiting, and abdominal pain at the lower right side of the abdomen. There was a collaboration with some European clinics to help diagnose a suspected genetic autoimmune inflammatory disease. Additionally, the patient's single parent, who travels to work outside of town, was invited to be present at the questioning of the caregiver, who confessed that she had a blank ampoule of pyrogenal, an immunomodulator, that she administered to induce symptoms in the patient; this was seized by the medical team. The mode of administration was by mouth, through food. The case was referred to Child Protective Services and managed according to their guidelines.

**Table 1:** Laboratory findings in the patient with suspected undifferentiated connective tissue disease

Laboratory findings	Results	Normal range
Hemoglobin	8.6 g/dL	11.0 - 14.5 g/dL
Leukocyte count	18,000 cells/μL	6,000 - 17,500 cells/μL
Erythrocyte sedimentation rate	149 mm/hour	< 10 mm/hour
C-reactive protein	267.9 mg/L	< 10 mg/L
Procalcitonin	269.5 ng/mL	< 2.0 ng/mL
Immunoglobulin M (IgM)	2.7 g/L	0.50 - 2.20 g/L

**Table 2:** Histopathological findings

Tests	Result
Trepanobiopsy	Excluded the diagnosis of leukemia
Skin and kidney immunohistochemical study	Confirmed mesangial proliferative glomerulonephritis with a weak tubule-interstitial component and secondary microvasculitis

## Discussion

This case illustrates the complexities of diagnosing and managing FDIA-associated illnesses in pediatric patients. It emphasizes the importance of considering FDIA in cases where unexplained or recurrent fevers are resistant to antipyretics and when caregivers exhibit concerning behaviors. Also, it underscores the need for a multidisciplinary approach to care. Given the complex nature of this case, the involvement of other specialties from different fields is relevant. Also, the discussion delves into the importance of early recognition, understanding the long-term impact, and the role of video surveillance.

Relevance of early recognition

A crucial aspect highlighted by the case is the need for early recognition of FDIA. Concerns about caregivers exhibiting concerning behaviors should prompt further evaluation or investigation. In cases where unexplained or recurrent fevers are resistant to antipyretics, consider FDIA. Pediatricians play a pivotal role in recognizing red flags and keenly advocating for the safety and well-being of vulnerable kids. Pediatricians should not consider the apparent intentions of the caregiver when there are clinical grounds to suspect FDIA, nor should they rule out the possibility of FDIA based on the caregiver’s psychiatric history [6]. Many pediatric deaths with undetermined causes may very well be due to FDIA [7]. There is a recognition that poor relationships with relatives and poor social networks constitute environmental risk factors for FDIA [8].

A multidisciplinary approach to care

The unique challenges that come with FDIA necessitate a multidisciplinary approach to care. The exact cause of FDIA, or MSBP, is not clear. However, experts say that biological and psychological factors play a role in the development of this disorder [9]. Various specialties, including pediatrics, social work, psychology, psychiatry, and child advocacy, must collaborate as each discipline contributes valuable insights and expertise.

Treatment, long-term impact, and psychological trauma

Additionally, this case raises questions concerning the long-term impact of FDIA on the victim. Beyond the medical consequences observed in this case, there may be long-lasting psychological trauma as a result of deception and manipulation perpetrated by caregivers that indicates ongoing intervention and support. In this case, the victim underwent medical treatment and psychotherapy. It is a must that the physicians involved refer to individual or family therapy [10].

The role of video surveillance

Video cameras set in place for caregivers suspected of FDIA are highly effective in identifying inappropriate behavior when considering early recognition and interventions [2]. It serves as an effective tool for identifying inappropriate behavior and corroborating suspicions. Video surveillance serves as objective evidence of caregiver actions, aiding in early recognition, and is a crucial measure in preventing FDIA or further harm to the victim and interrupting the cycle of abuse.

## Conclusions

Factitious disorder imposed on another is a challenging diagnosis that requires keen attention, awareness, and a multidisciplinary approach. Early recognition and intervention are critical to avoid further harm to the victim. This case underscores the significance of vigilance in recognizing potential cases of FDIA and advocating for the safety and well-being of pediatric patients. Also, video surveillance is crucial in detecting and preventing FDIA. Finally, beyond medical management, individual or family therapy is a must.
